# Rank among Peers during Game Competition Affects the Tendency to Make Risky Choices in Adolescent Males

**DOI:** 10.3389/fpsyg.2017.00016

**Published:** 2017-01-24

**Authors:** Jerome C. Foo, Kohei Nagase, Sawako Naramura-Ohno, Kazuhiro Yoshiuchi, Yoshiharu Yamamoto, Kenji Morita

**Affiliations:** ^1^Physical and Health Education, Graduate School of Education, The University of TokyoTokyo, Japan; ^2^Department of Stress Sciences and Psychosomatic Medicine, Graduate School of Medicine, The University of TokyoTokyo, Japan

**Keywords:** adolescence, peer influence, risk taking, competition, smartphone

## Abstract

It has been shown that adolescents take more risks when they are with peers than when they are alone, presumably because the presence of peers can be a social reward/punishment that can bias decision making. Competition is inherent in peer interactions, and recent work has demonstrated that winning/losing is an intrinsic social reward/punishment. Taken together, it can be hypothesized that competition amongst peers affects adolescents’ risky behavior. While there is much evidence that status amongst peers can relate to antisocial/aggressive behavior, it remains unclear whether risky behavior is affected. Moreover, the degree to which ‘temporary status,’ such as ranking in a short-term competitive game, affects behavior is uncertain, an important issue because adolescents might be sensitive to situations or factors which potentially destabilize existing hierarchies. In this experiment, these issues were directly explored in the classroom environment using smartphone technology and Wi-Fi setup. Male junior high school students (aged 14–15) performed a roulette game task on smartphones, playing either independently or against five classmates. In the latter case, the students’ current ranks within the group of six were constantly presented on smartphone screens. To dissociate the effects of the students’ reactions to ranks from their actual performances, unknown to the students, the ranks presented were actually predetermined so that about half of the students were continuously presented with high ranks whereas the other half were continuously presented with low ranks. We found that the students presented with low ranks made more risky plays than those not presented with ranks or those presented with high ranks. This result suggests that even temporary status significantly affects adolescents’ risky behavior, and also demonstrates the usefulness of smartphones in examining and manipulating peer interactions in classroom experiments.

## Introduction

It has been shown that adolescents are inclined to engage in risky behavior, potentially resulting in serious problems (Steinberg, 2008; Somerville and Casey, 2010; Reyna and Brainerd, 2011; Defoe et al., 2015), and it has also been found that this tendency for risky behavior is enhanced by the presence of peers (Steinberg, 2008). Specifically, adolescents are more likely to make risky decisions (Gardner and Steinberg, 2005; Smith et al., 2014) and are more sensitive to immediate reward (O’Brien et al., 2011; Weigard et al., 2014) when knowing that they are being observed by peers than when they are alone. It has been suggested that these effects appear because the presence of peers can constitute a social reward that can bias decision making, presumably through the modulation of the reward system in the brain (Chein et al., 2011).

It is striking that even the mere presence of peers can affect risky behavior (Gardner and Steinberg, 2005). Naturally, when peers are present, they generally also interact with one another, making it important to examine the effects of peer interactions in detail. Indeed, a recent study has shown that peer observation and peer advice have distinguishable effects on risky decision making (Haddad et al., 2014). Competition is another typical type of peer interaction. Recent work (in adults) has shown, through fitting of the behavior in an auction task by reinforcement learning models, that winning/losing a competition constitutes an intrinsic social reward/punishment, independent of monetary outcome, while also suggesting neural and hormonal substrates (van den Bos et al., 2013a,b). Taken together with the abovementioned suggestion that peer presence affects risky behavior by modulating the reward system, we hypothesized that awareness of one’s place in competitive interactions amongst peers would further modulate the reward system, and potentially affect risky behavior more. It was difficult, however, to specify exact expectations of the possible effects from the studies introduced above; we discuss this issue in relation to a wider behavioral economics literature in the Section “Discussion.”

Much evidence shows that peer status or hierarchies can be related to antisocial or aggressive behavior in adolescents and children (Coie et al., 1982; Xie et al., 2002; Prinstein and Cillessen, 2003). However, it remains unclear whether and how risky decision making is affected by these interpersonal dynamics. While previous studies typically examine established hierarchies and their long term changes, it is an open question whether and how temporary status, such as ranking in a competitive game, can affect behavior. This is an important issue to examine as adolescents might be more sensitive to temporary status changes than adults given their potential to destabilize existing hierarchies, resulting in the higher levels of risky behavior observed. To date, these temporary status changes and their effects have been technically challenging to examine in their real world environments (i.e., the classroom). In the present work, we directly tackle this issue using the advantages offered by smartphone devices.

Specifically, we examined risky decision making behavior in adolescents in the classroom environment where they were surrounded by their actual peers. A total of 131 junior high school male students (aged 14–15 years old) performed a roulette task, similar to the one used in a previous study (Smith et al., 2014), implemented on smartphone devices. Participants were presented with a series of roulette wheels showing the probability of gain and loss, and decided whether to ‘play’ or ‘pass’ for each wheel. Half of the students performed the task independently while the other half played against five unidentified classmates. In the latter case, students’ current ranks within their group of six were continuously presented on the smartphone displays. In order to dissociate the effects of the students’ reactions to ranks from their actual performances, the ranks presented were not real: unbeknownst to the students, ranks were predetermined so that about half of the students were continuously presented with fluctuating high ranks whereas the other half were continuously presented with fluctuating low ranks. Students were led to believe that ranks were calculated in real time through Wi-Fi networks set up in the classrooms at the time of the experiment.

We hypothesized that the real-time presentation of ranks amongst peers would modulate participants’ tendencies to make risky choices, or more specifically, that presentation of low ranks, or high ranks, or both would increase the ratio of choosing ‘play’ especially for roulette wheels with non-positive expected values (EVs: *X* × *P*_gain_(*X*) - *Y* × *P*_loss_(*Y*), where *X* and *Y* are the point amounts that can be gained or lost and *P*_gain_(*X*) and *P*_loss_(*Y*) are the probabilities of gain and loss, respectively; the EV of a given roulette wheel is approximately the average point amount obtained by playing that wheel many times). A previous study using a similar roulette task (Smith et al., 2014) showed that the presence of peers increases the ratio of choosing ‘play’ for roulette wheels with negative EVs, and called this an increase of risk-taking caused by the presence of peers. This usage of the word ‘risk’ includes the meaning ‘disadvantageous,’ and differs from the usage of (pure) ‘risk’ in the fields of behavioral economics, where (pure) ‘risk’ refers to the variance/variability of outcomes and is conceptually orthogonal to EV (c.f., Defoe et al., 2015). According to the definition of pure risk in behavioral economics, ‘play’ for the roulette wheel with EV = 0 is riskier than ‘pass,’ while ‘playing’ on roulette wheels with negative [positive] EVs is both riskier and more disadvantageous [advantageous] (in terms of EV) than ‘pass.’ Considering these, outcomes of all roulette wheels are examined but we attend specifically to ‘play’ ratios for roulette wheels with non-positive EVs.

## Materials and Methods

### Participants

Data was collected from a group of 131 participants (adolescent male students, aged 14–15 years old) made up of three whole classes (45, 42, and 44 students) in the 3rd year of a junior high school in Osaka, Japan. One week prior to the start of the experiment, parents of students were distributed a document via students about the voluntary nature of the study and its contents (including the presentation of manipulated information related to relative ranks of accuracy rates, but without explanatory details about what they might be) and given the option to withdraw their children from participation if they so desired (none did so). On the day of the experiment, the voluntary nature of the study and the contents of the experiment (excluding the falseness of the presented ranks) were explained to participants and written informed consent was obtained from all participants. Students were told that the outcomes of the experiment would not affect their school grades in any way. Participants were not paid for their participation in the experiment. The research ethics committee at the University of Tokyo approved of the present study.

### Roulette Task Paradigm

**FIGURE 1:**
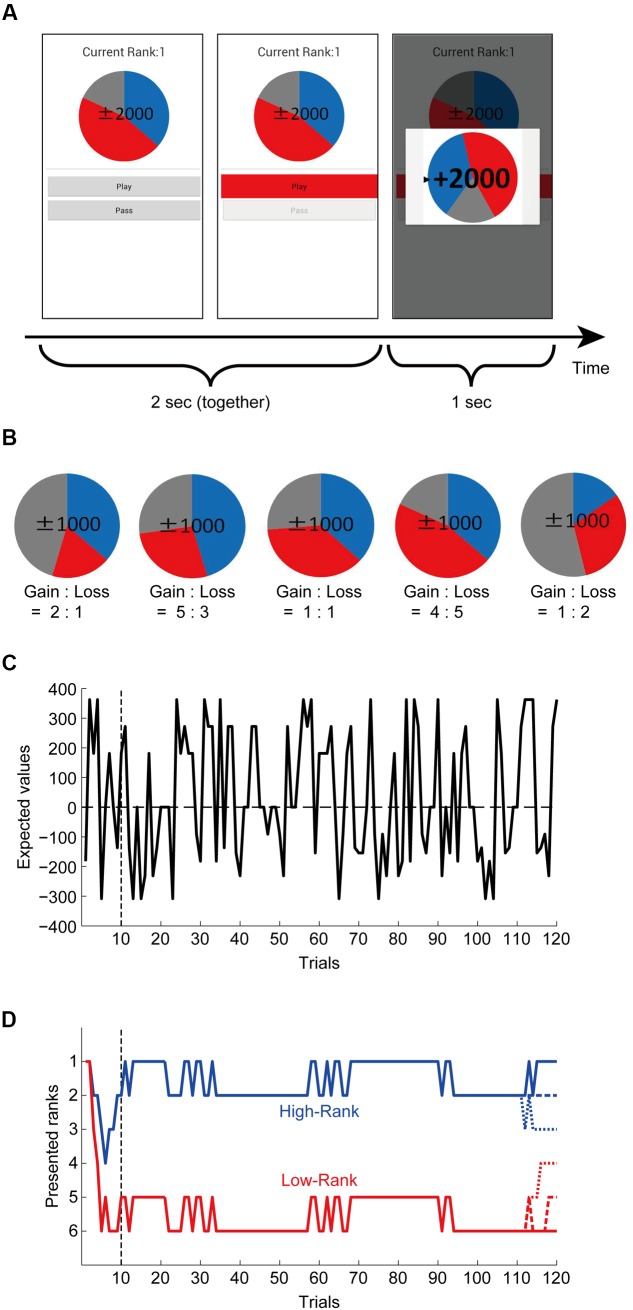
**Roulette task and rank presentation. (A)** Task paradigm. Participants chose to either ‘play’ or ‘pass’ (by pressing a corresponding button) based on information visualized by a color-coded roulette wheel. The number of points at stake each trial was indicated in the center of the roulette wheel, while the different colors indicated probability of gain/loss (blue = gain, red = loss, gray = zero). There were also participants who performed the task independently without rank presentation: ‘Current Rank’ was not presented for them. **(B)** Five different configurations of the gain/loss probabilities of roulette wheels. The point amounts which could be gained or lost on each trial by playing were 1000, 1500, 2000, or 0 for each of the five probability configurations (shown are the cases with 1000 points). **(C)** Expected values (EVs) of the presented roulettes. **(D)** Pre-determined false rank patterns. There were two grand-patterns: one was continuously at high ranks (1st or 2nd) and the other was continuously at low ranks (5th or 6th) except for the initial and last ∼10 trials. For the last ∼10 trials, both the high-rank and low-rank grand-patterns further differentiated into three sub-patterns ending with 1st, 2nd, or 3rd rank and 4th, 5th, or 6th rank, respectively, as shown in the figure.

We implemented a roulette risky decision making task similar to the one used in prior work (Smith et al., 2014) in smartphone devices (described in detail below). Participants chose to either ‘play’ or ‘pass’ on virtually spinning a roulette wheel based on information displayed on a color-coded roulette wheel graphic (**Figure 1A**, left panel). The number of points at stake each trial was indicated in the center of the roulette wheel, while the different colors on the wheel indicated probability of gain/loss (blue = gain, red = loss, gray = zero). There were five different configurations of gain/loss probabilities (**Figure 1B**, **Table 1**). In this experiment, the exact probabilities were not displayed to promote intuitive decision making rather than explicit calculation of the EVs (and also to control for potential individual differences in calculation ability). The point amounts which could be gained or lost on each trial by playing were: 1000, 1500 and 2000 and 0 (for each of the five probability configurations), resulting in a variety of EVs (**Figure 1C**, **Table 1**). If ‘pass’ was selected, 0 points would be obtained on that trial.

**Table 1:** Probabilities of gain and loss, and variations in the expected values (EVs), for each roulette wheel.

	Roulette-wheel with gain: loss = 2 : 1 (36% : 18%)	Roulette-wheel with gain: loss = 5 : 3 (45% : 27%)	Roulette-wheel with gain: loss = 1 : 1 (37% : 37%)	Roulette-wheel with gain: loss = 4 : 5 (36% : 46%)	Roulette-wheel with gain: loss = 1 : 2 (15% : 31%)
Points at stake ± 1000	181	181	0	-91	-154
Points at stake ± 1500	272	272	0	-137	-231
Points at stake ± 2000	362	362	0	-182	-308

On each trial, participants were given 2000 ms to choose whether to play or pass upon which the button they pressed was highlighted in red (**Figure 1A**, middle panel). The task was synchronized across participants; regardless of response time, the result was not shown until the full 2000 ms had passed. Results of the trial were then displayed for 1000 ms (**Figure 1A**, right panel), along with a graphic showing the final position of the roulette wheel. This was followed immediately by the beginning of the subsequent trial. Participants were instructed to do their best to maximize their point totals. If no responses were recorded during 2000 ms response window, choice options were grayed out until the next trial. Participants were told that there was a penalty for failing to respond in time, and time-out trials were excluded from the analysis. The total number of accumulated points was displayed at the end of the session.

The experimental session was comprised of 120 trials, lasting ∼6 min. The trial order was the same across all participants. A 20-trial training session preceded the main experiment to get participants accustomed to the task.

### Presentation of Rank

All participants in each class performed the task at the same time. Participants were grouped into ‘No-Rank’ and ranked groups. Participants in the ‘No-Rank’ group played the task with no rank displayed. Ranked participants ‘competed’ in groups of 6 but were unaware of the identities of their ‘competitors.’ In the ranked group, ranks were displayed on their smartphone screen throughout the experimental session; unknown to these students, ranks were actually predetermined and fluctuated over the experiment based on a preset schedule. This schedule and outcomes of trials were manipulated so that changes in rank appeared as natural and plausible as possible. As such, we included ‘gain’, ‘loss’, and ‘zero’ sections for all roulette wheels (**Figure 1B**) (i.e., even if the participants ‘passed’ or got a score of 0 on a given trial, their rank could change in either direction if competitors ‘played’).

Ranks were manipulated such that the ranked group contained two sub-groups: ‘High-Rank’ and ‘Low-Rank.’ For those in the ‘High-Rank’ group, ranks were made to fluctuate between 1st and 2nd place. For those in the ‘Low-Rank’ group, the displayed ranks fluctuated between 5th and 6th place. To make the initial establishment of ranks seem as natural as possible, ranks during the first 10 trials were made to fluctuate with more frequency before reaching the established high or low rank. During the final 10 trials leading up to the end of the session, ranks were set to change such that all ranks from 1 to 6 would all accounted for (**Figure 1D**). That is, those in the ‘High-Rank’ group would finish the session ranked 1st, 2nd, or 3rd, while those in the ‘Low-Rank’ group would end the session ranked 4th, 5th, or 6th.

A number of steps were taken to ensure that students did not realize that ranks were false. Colored stickers were used to mark the smartphones of ‘competing’ groups (students with the same colors competed with each other) but smartphones were shuﬄed when they were distributed so that a single group was dispersed within the classroom. This was done to discourage the students from looking at the devices of the students around them and instead to focus on their own game, while also allowing us to keep track of the ‘competing’ groups. Next, we instructed participants to place the devices flat on their desks, not to look at other persons’ smartphones, and not to communicate during the experiment. Screen brightness of devices was set at minimum so that screens could not be easily seen from the side. In addition, we set up a laptop computer and Wi-Fi routers at the rear of the classrooms (but they were not actually used).

We originally planned to have 24 students in the ranked group per class (four groups of six students) with the remainder of students in each class being in the ‘No-Rank’ group. Due to practical issues in device preparation and delivery, in some cases there were less than six students in a single ‘competing’ group; however, students were not explicitly told this and were presumably unaware of it as the seats of the students in each competing group were dispersed as mentioned above. In the end, 65 students were in the ‘No-Rank’ group while 36 students were in the ‘High-Rank’ group, and 30 students were in the ‘Low-Rank’ group.

### Experimental Device and Application

Experiments were carried out on Covia (CP-D02) Android smartphones (Covia Inc., Yokohama, Japan) running Android Version 4.0. A custom-made application for presenting psychological tasks was used. The smartphone application was designed to be always full-screen such that participants could not view the device settings and the devices were distributed with the application already started. During the task, menu buttons were disabled to prevent any errant presses.

### Statistical Analyses

Statistical analyses were conducted using MATLAB (MathWorks Inc., Natick, MA, USA), including its Statistics Toolbox / Statistics and Machine Learning Toolbox, the codes in the file http://www.sbirc.ed.ac.uk/cyril/glm/GLM_lectures.html, which is a companion to the lectures by Cyril Pernet, and the codes formerly in http://rnpsychology.org/ (by Ryosuke Niimi), and also R^1^. The level of statistical significance was set at *p* < 0.05. For each individual participant, we calculated the ratio of ‘play’ (as defined above) for trials that met particular criteria for each analysis (described in the Results), and compared the across-participant distributions of the ‘play ratio’ in the three rank groups (No-Rank, High-Rank, and Low-Rank). For the effect size measure, we adopted Keselman and colleagues’ *d*_j_ (see below) (Keselman et al., 2008; Peng and Chen, 2014), which applies when normality is confirmed but variances are unequal and sample sizes are unequal. (instead of the widely used Cohen’s *d* which assumes both normality and homogeneity). To be stringent, we examined all values of *d*_j_ to get an accurate picture of the effect size (Keselman et al., 2008; Peng and Chen, 2014).

Keselman and colleagues′d_j_

$$d_{j}=\frac{{\overset{-}{X}}_{1}-{\overset{-}{X}}_{2}}{{\overset{\wedge }{\sigma }}_{j}},\;where$$

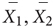
 = sample means of groups 1 and 2, respectively, and


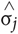
 = sample standard deviation of either groups 1 or 2.

In addition to the analyses based on the play ratio, we explored sensitivity to differences in EVs in individual trials (11th to 120th, excluding the initial 10 trials) using a Generalized Estimating Equation (GEE) (Liang and Zeger, 1986) analysis in reference to an approach used in a previous study (Weller et al., 2015). The GEEQBOX toolbox for MATLAB (Ratcliffe and Shults, 2008)^2^ was used for GEE analyses. EVs were first calculated for each experimental trial: probabilities of gain and loss denoted by roulette wheels (approximate proportions of colored pixels for gain + loss were extracted from roulette wheel images and multiplied by gain/loss ratios) were multiplied by presented point amounts and then summed (**Figure 1C**; **Table 1**). Explicit probabilities were not displayed as it was undesirable to have participants calculating EVs during the task. Outcomes were modeled as ‘play’ = 1 and ‘pass’ = 0, and the Bernoulli distribution assumption was used. As for correlation structure, the Markov correlation structure was assumed. Covariates of trials (11th to 120th), normalized EVs [=(EVs - mean across 120 trials)/(standard deviation across 120 trials)], High-Rank (dummy-coded, i.e., 1 for High-Rank and 0 for No-Rank or Low-Rank), Low-Rank (dummy-coded, i.e., 1 for Low-Rank and 0 for No-Rank or High-Rank), interaction terms between normalized EVs × High-Rank and normalized EVs × Low-Rank, and a constant term were specified in the model. GEEQBOX provides two versions of results: a robust covariance matrix-based one and a model-based covariance matrix-based one. We present the former because although the Markov structure appeared to be a natural assumption, it was not known to be the best assumption.

In the GEE analysis described above, High-Rank and Low-Rank were individually dummy-coded, and therefore a direct comparison between High-Rank and Low-Rank, as well as detection of a possible interaction between the Low vs. High Ranks and EVs, could not be achieved. In order to achieve these, we conducted a separate GEE analysis on the data of High-Rank and Low-Rank participants only (i.e., excluding the No-Rank participants), in which covariates of trials (11th to 120th), normalized EVs, Low-Rank (vs. High-Rank) (dummy-coded, i.e., 1 for Low-Rank and 0 for High-Rank), interaction term between normalized EVs × Low-Rank (vs. High-Rank), and a constant term were specified in the model. Similarly to the original GEE analysis described above, the Markov correlation structure was assumed, and the robust covariance matrix-based results are reported.

### Additional Notes

Participants were given instructions by the experimenters, along with a teacher from the school. All participants completed a mental health questionnaire. The experiment described here was part of a larger experiment at the junior high school; before performing the roulette task, participants performed a different reinforcement learning task twice (i.e., two sessions), once with ‘false’ rank presentation and once with no rank, for which results are not described in this report. It was our original intention to also conduct two sessions of the roulette task, but due to class period length and time considerations we were only able to conduct a single session, resulting in the current design. Given these constraints and as it was considered ethically desirable, participants presented with low rank in the roulette task were presented with high rank in the reinforcement learning task, either in the first or second session, and vice versa (except for one student who was not presented with ranks in the reinforcement learning task due to a technical issue) so that there was no student who was presented with only low ranks in both tasks. For the purpose of the present study, it would have been ideal if we could counterbalance the possible effects of rank presentation in the preceding task in such a way that a half of participants presented with high ranks in the roulette task experienced low ranks in the preceding task while the other half experienced high ranks in the preceding task, but we could not do so because of ethical and practical reasons. Participants presented with no rank in the roulette task were also presented with a rank in the preceding task, either high or low ranks in either the first or second session, and the data of these participants were used to examine possible effects of the rank presentation in the preceding task (i.e., the reinforcement learning task) on the behavior in the roulette task (described in the Results).

Although ranks for experiments were false, scores obtained by each student were reflective of their true behavior. It is interesting to note that even though students did not know to which six person group they belonged, after the experiment was over, they compared their scores (and ranks where applicable) with people seated close to them without prompting, showing a natural desire to confirm their positions amongst their peers. The fact that the rank was false was explained to the students by their teacher in a debriefing session several days after the main experiment. According to the teacher who conducted the debriefing sessions, the students believed that the ranks had been real and remarked that without the debriefing, would not have realized that ranks shown during the experiment were fake (indicating that even though it might have been possible for the students to read the document for their parents including description about the experimental manipulation of presented ranks, they did not do so or at least did not well understand the meaning of the description with respect to the task).

## Results

**FIGURE 2:**
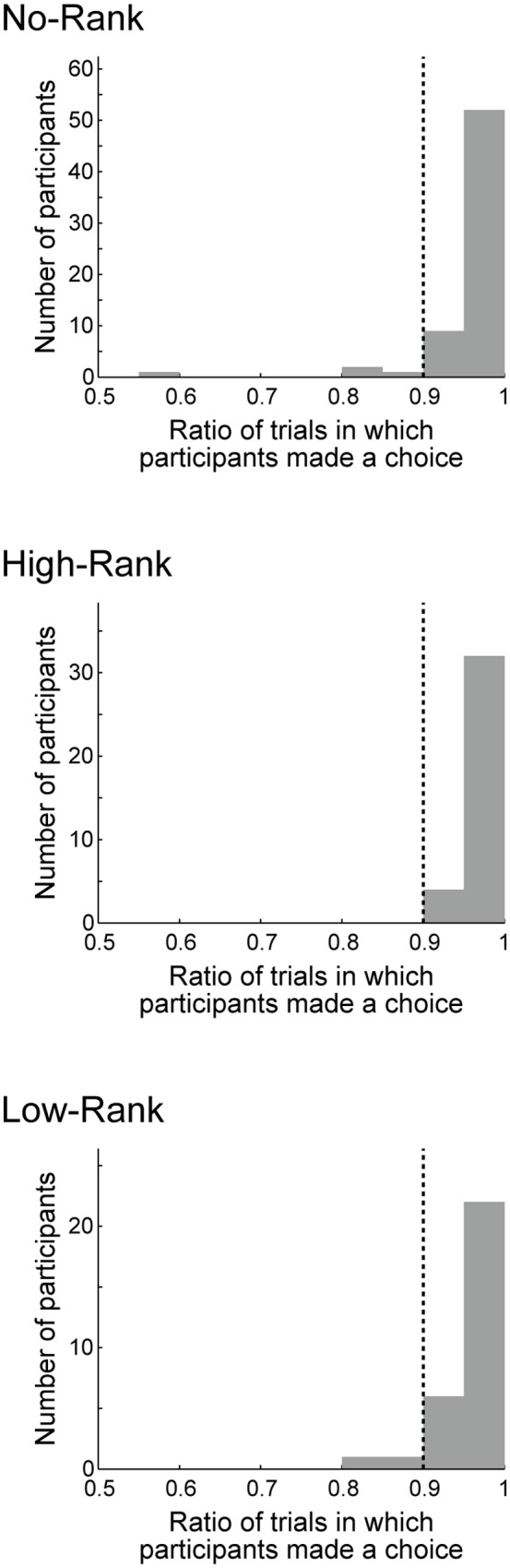
**Histograms of the ratio of trials in which participants made a choice, either ‘play’ or ‘pass,’ within the time limit (2 s)**. The three panels show the histograms for the group without rank presentation (No-Rank), with high-rank presentation (High-Rank), and with low-rank presentation (Low-Rank). The vertical dotted lines in each panel indicate the 90% line: the small number of participants (four students in the No-Rank group and two students in the Low-Rank group) to the left of this line were excluded from the subsequent analyses.

The majority of participants in all three rank groups made their choices (‘play’ or ‘pass’), within the time limit (2 s) in ≥90% of the trials (**Figure 2**). The remaining participants (four students in the No-Rank group and two students in the Low-Rank group) were excluded from subsequent analyses.

**FIGURE 3:**
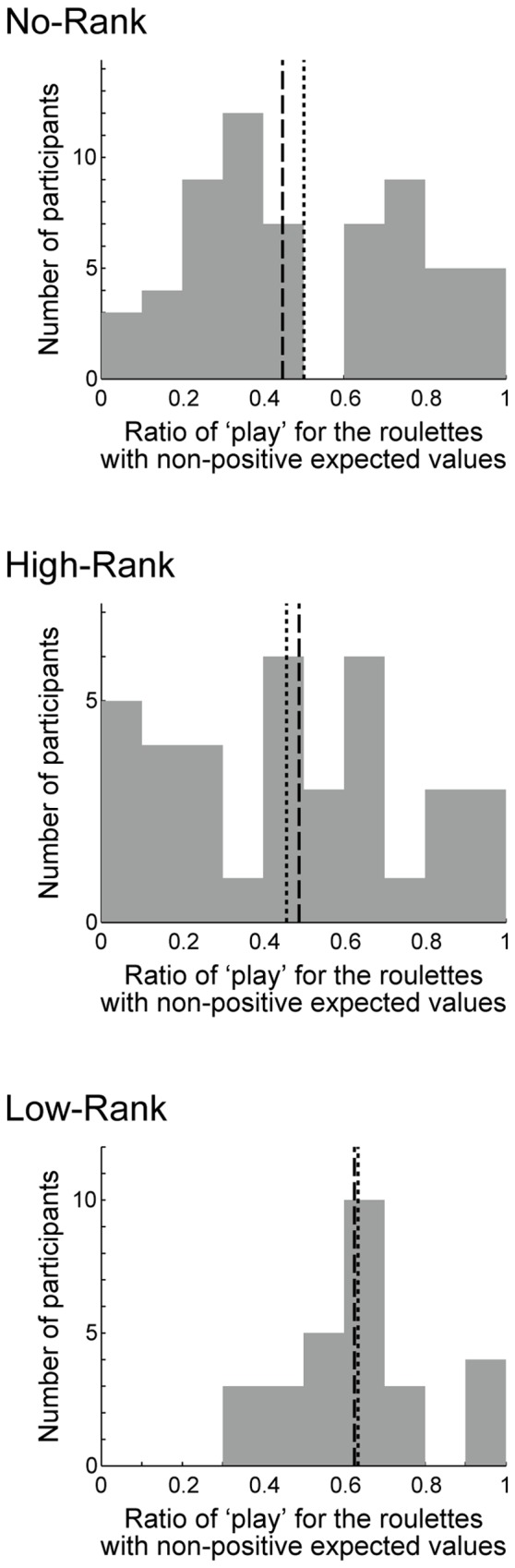
**Histograms of the ratio of choosing ‘play’ in the trials in which a roulette wheel with non-positive expected value (the right three of **Figure 1B**) was presented and a choice was made, excluding the initial 10 trials during which the presented ranks were gradually stabilized to either high or low ranks**. The three panels show the histograms for the three rank groups. The dotted lines indicate the means: 0.501 (No-Rank), 0.458 (High-Rank), and 0.634 (Low-Rank). The standard deviations were 0.271 (No-Rank), 0.289 (High-Rank), and 0.175 (Low-Rank). The dashed lines indicate the medians: 0.448 (No-Rank), 0.489 (High-Rank), and 0.626 (Low-Rank).

For each participant, we calculated the play ratio in the trials in which roulette wheels with non-positive EVs (the right three of **Figure 1B**) were presented and a choice was made. The initial 10 trials during which presented ranks gradually stabilized to either high or low ranks were excluded from analysis (**Figure 1D**). In order to test the effects of rank presentation on risk taking, we compared the play ratio between the three rank groups (**Figure 3**) by conducting one-way ANOVA, finding a significant effect [*p* = 0.0217, η^2^ = 0.0609 (95% confidence interval: lower: 0.0006, upper: 0.1480)] as hypothesized. Since the Bartlett test of homogeneity of variances revealed significant inhomogeneity (*p* = 0.02295), we also conducted Welch’s test (Welch, 1951) (Welch’s ANOVA), which does not assume equal variances, and found that the result was also significant (*p* = 0.003781). Pairwise Welch’s *t*-tests with Bonferroni correction (corrected significance level *α* = 0.0166) revealed significant differences between High-Rank and Low-Rank (*p* = 0.003724, Keselman and colleagues’ *d*_1_ = 0.610, *d*_2_ = 1.009) and between No-Rank and Low-Rank (*p* = 0.006645, Keselman and colleagues’ *d*_1_ = 0.493, *d*_2_ = 0.763), but no difference between No-Rank and High-Rank (*p* = 0.4713, Keselman and colleagues’ *d*_1_ = 0.148, *d*_2_ = 0.158). As the effect size measure, we adopted Keselman and colleagues’ *d*_j_, because normality was confirmed but variances were unequal and sample sizes were unequal (Keselman et al., 2008; Peng and Chen, 2014). Even if we take the smaller *d*_j_, effect sizes were not small between High-Rank and Low-Rank, and between No-Rank and Low-Rank.

In each of the No-Rank and High-Rank groups, there were two (in total four) participants whose ratio of ‘play’ was 0 and one (in total two) whose ‘play’ ratio was 1, while there was no such participant in the Low-Rank group. Excluding these participants (in total six) did not largely change the results described above: one-way ANOVA revealed a significant effect [*p* = 0.0225, η^2^ = 0.0634 (95% confidence interval: lower: 0.0005, upper: 0.1538)], Welch’s ANOVA also revealed a significant effect (*p* = 0.006063), and pairwise Welch’s *t*-tests with Bonferroni correction found significant differences between High-Rank and Low-Rank [*p* = 0.004921, (Keselman and colleagues’) *d*_1_ = 0.629 and *d*_2_ = 0.944] and between No-Rank and Low-Rank (*p* = 0.009391, *d*_1_ = 0.494 and *d*_2_ = 0.713), but no difference between No-Rank and High-Rank (*p* = 0.4779, *d*_1_ = 0.153 and *d*_2_ = 0.159). These six participants were re-included in the analyses described below, because it is uncertain whether excluding them is valid or not given the nature of the task.

**FIGURE 4:**
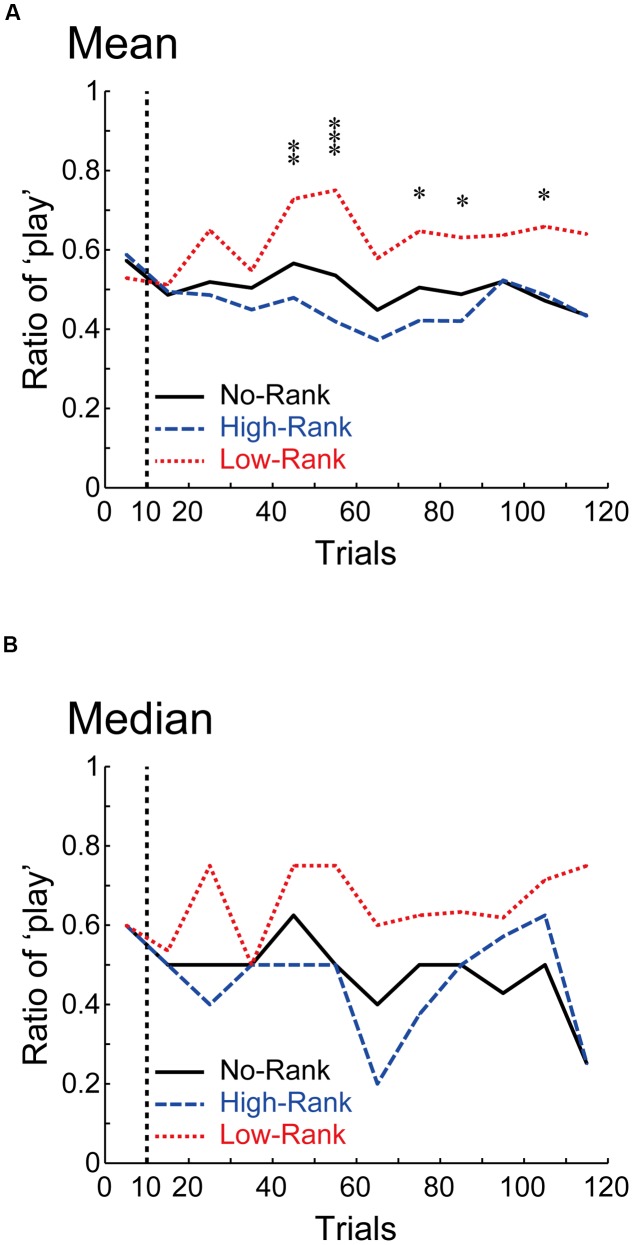
**How the ratio of ‘play’ for the non-positive roulette wheels changed along with trials. (A,B)** show the means and the medians, respectively. ^∗∗∗^*p* < 0.001, ^∗∗^*p* < 0.01, and ^∗^*p* < 0.05 [one-way ANOVA for each bin (including 10 trials), uncorrected for multiple comparison].

**Figure 4** shows how the play ratio for the non-positive roulette wheels changed as trials progressed (**Figure 4A**: mean, **Figure 4B**: median). As shown in the figure, once the presented ranks stabilized at either high or low rank (at around the 10th trial, **Figure 1D**), the play ratios in the three rank groups started to diverge, and these differences were then kept rather stably. This excludes the possibility that differences between the rank groups appeared only at the end of the task. Indeed, results of the aforementioned tests were not largely changed when the last 10 trials (in addition to the initial 10 trials) were excluded: one-way ANOVA revealed a significant effect (*p* = 0.0225, η^2^ = 0.0603 (95% confidence interval: lower: 0.0005, upper: 0.1473)) and Welch’s ANOVA also revealed a significant effect (*p* = 0.004514), and pairwise Welch’s *t*-tests with Bonferroni correction revealed significant differences between High-Rank and Low-Rank (*p* = 0.003814, Keselman and colleagues’ *d*_1_ = 0.611 and *d*_2_ = 0.995) and between No-Rank and Low-Rank (*p* = 0.008373, *d*_1_ = 0.483 and *d*_2_ = 0.733), but no difference between No-Rank and High-Rank (*p* = 0.4354, *d*_1_ = 0.172, *d*_2_ = 0.160).

**FIGURE 5:**
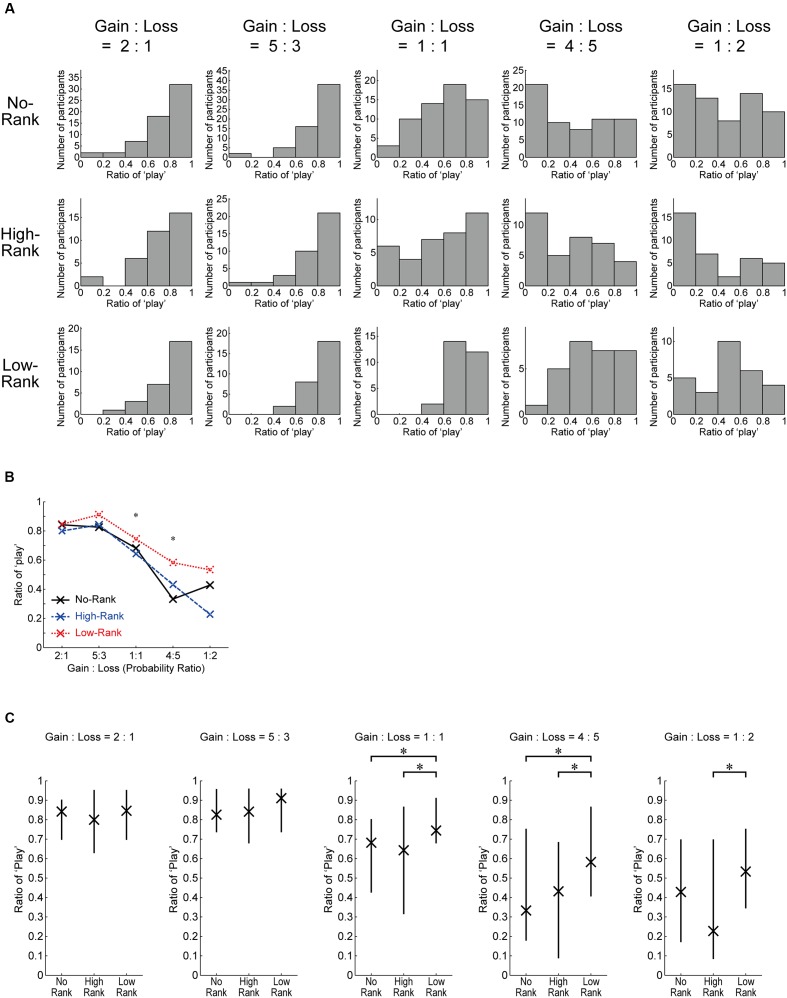
**Detailed data of the ratio of ‘play’ (excluding the initial 10 trials) for each roulette wheel in each rank group. (A)** Histograms. **(B)** Medians. ^∗^*p* < 0.05 (Kruskal–Wallis *H*-test, uncorrected for multiple comparison). **(C)** Medians and quantiles. ^∗^*p* < 0.05 (Wilcoxon rank-sum test (Mann–Whitney *U*-test), uncorrected for multiple comparison: for the 1:1 roulette, No-Rank vs. High-Rank (*p* = 0.7367, *r* = 0.0341), No-Rank vs. Low-Rank (*p* = 0.0134, *r* = 0.2621), and High-Rank vs. Low-Rank (*p* = 0.0184, *r* = 0.2948); for the 4:5 roulette, No-Rank vs. High-Rank (*p* = 0.6839, *r* = 0.0414), No-Rank vs. Low-Rank (*p* = 0.0140, *r* = 0.2605), and High-Rank vs. Low-Rank (*p* = 0.0205, *r* = 0.2896); for the 1:2 roulette, No-Rank vs. High-Rank (*p* = 0.1566, *r* = 0.1438), No-Rank vs. Low-Rank (*p* = 0.2225, *r* = 0.1293), and High-Rank vs. Low-Rank (*p* = 0.0363, *r* = 0.2617).

**Figure 5A** shows histograms of the play ratio (excluding the initial 10 trials) for each roulette wheel in each rank group. **Figure 5B** shows the medians, and **Figure 5C** shows the medians and quantiles. As shown in **Figure 5A**, whereas the shape of the distribution is largely similar across the rank groups for the roulette wheels with positive EVs, the shape appears to differ depending on the rank groups for the non-positive roulettes. Indeed, the Kruskal–Wallis *H*-test revealed significant differences between the rank groups for the 1:1 and 4:5 roulettes (**Figure 5B**), and the Wilcoxon rank-sum test (Mann–Whitney *U*-test) revealed significant differences between High-Rank and Low-Rank for the three non-positive roulettes and also between No-Rank and Low-Rank for the 1:1 and 4:5 roulettes (**Figure 5C**); non-parametric tests were used here because some of the distributions prominently deviated from a normal distribution as seen in **Figure 5A** and also in the results of the Kolmogorov–Smirnov test (**Table 2**).

**Table 2:** *p*-values for Kolmogorov–Smirnov test for normality of the across-participant distributions of the ratio of ‘play’ for each roulette-wheel type.

l	Roulette-wheel with gain: loss = 2 : 1	Roulette-wheel with gain: loss = 5 : 3	Roulette-wheel with gain: loss = 1 : 1	Roulette-wheel with gain: loss = 4 : 5	Roulette-wheel with gain: loss = 1 : 2
lNo-Rank	0.05392	0.06438	0.2131	0.04771	0.362
lHigh-Rank	0.3003	0.2103	0.6941	0.6308	0.2178
lLow-Rank	0.1797	0.0611	0.6486	0.9547	0.9

**FIGURE 6:**
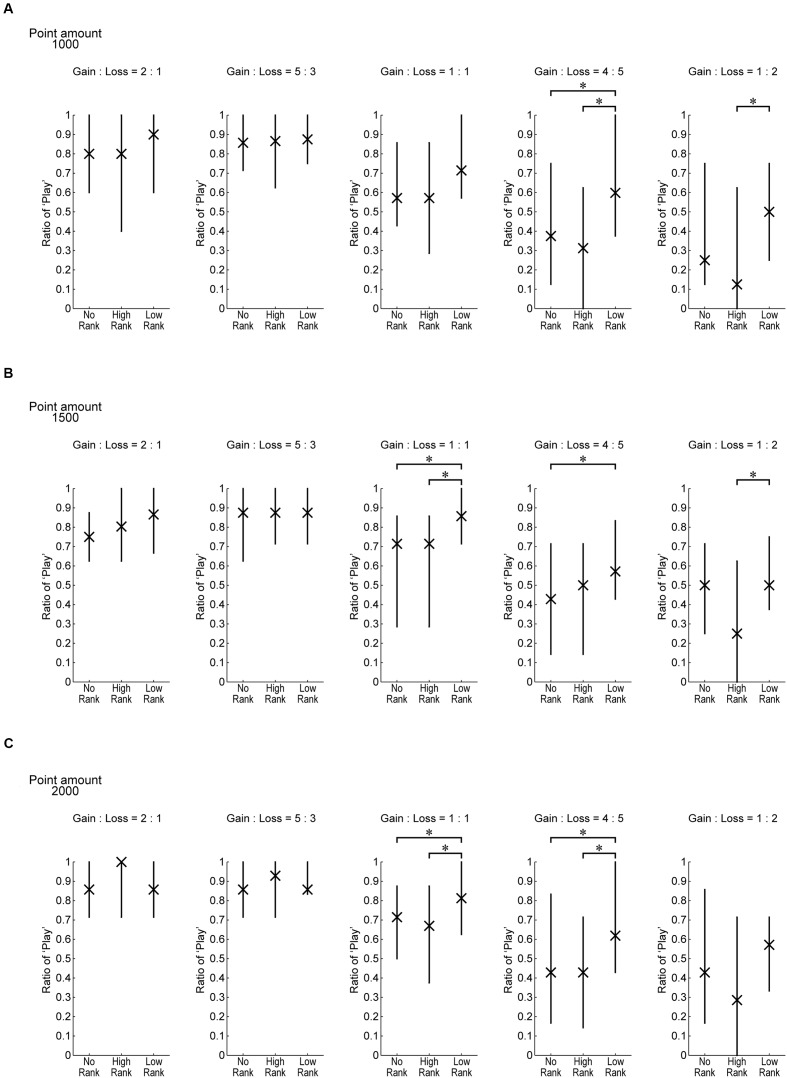
**The same data as **Figure 5C** separately for the three possible point amounts which could be gained or lost on each trial by playing**. ^∗^*p* < .05 (Wilcoxon rank-sum test (Mann–Whitney *U*-test), uncorrected for multiple comparison).

**Figure 6** plots the same data as **Figure 5C**, separately showing the play ratios for when the three different point amounts were at stake. Overall tendencies appear to be similar regardless of the point amount.

**FIGURE 7:**
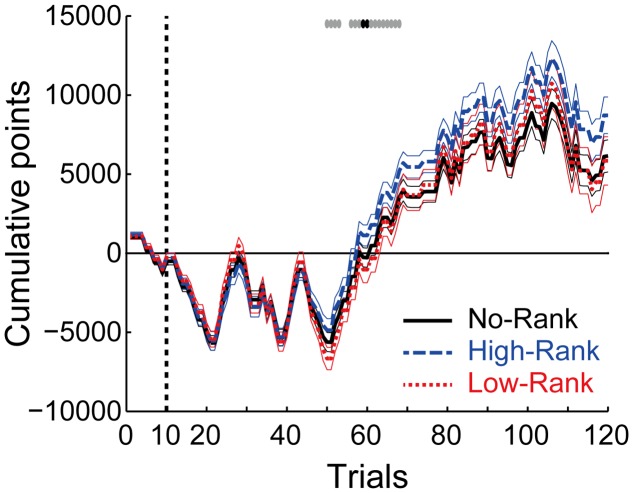
**Cumulative points along with trials in the three rank groups**. The thick lines and thin lines indicate the mean ± standard error for each group. The top gray and black symbols indicate trials where there exists a tendency of difference (*p* < 0.10) or a significant difference (*p* < 0.05), respectively, between High-Rank and Low-Rank participants (*t*-test, uncorrected for multiple comparison).

**Figure 7** shows the cumulative points in the three rank groups. As shown in the figure, during the middle of the task, the average cumulative points diverged between High-Rank and Low-Rank participants, with the former obtaining more points than the latter. Since all the groups played exactly the same roulette wheels, this disparity likely originates from differences in the play ratio of roulette wheels with non-positive EVs: in an effort to get more points, Low-Rank participants played non-positive roulette wheels more frequently than High-Rank participants, thereby losing more points. It is interesting to note that although the ranks presented were not real, at the group level, rank appears to have played a part in determining outcomes, in a ‘self-fulfilling prophecy’ fashion. This observation might suggest a top–down effect of rank, with higher rank being a reinforcer while lower rank is a punishment. It may appear inconsistent that the cumulative points of Low-Rank participants did not go below those of No-Rank participants, despite the differing play ratio for non-positive roulette wheels between these two groups as described above. This can be explained by the fact that the difference in the play ratio between No-Rank and Low-Rank groups was generally smaller than the difference between High-Rank and Low-Rank groups (**Figure 4**), and especially so for the most disadvantageous (Gain: Loss = 1: 2) roulette wheel (**Figures 5** and **6**).

**Table 3** shows the results of the GEE analysis (Liang and Zeger, 1986; Ratcliffe and Shults, 2008; Weller et al., 2015) for the sensitivity to differences in EVs in individual trials (11th to 120th, excluding the initial 10 trials). A significant main effect of (normalized) EV was observed, with higher likelihood to ‘play’ as EV increased, i.e., as the roulette wheel became more favorable. A main effect of Low-Rank was also significant: Low-Rank increased the probability of ‘playing’. No significant effect of trials, High-Rank, and no interactions between (normalized) EV and ranks were observed. **Table 4** shows the results of a separate GEE analysis for the data of Low-Rank and High-Rank participants only (i.e., excluding the data of No-Rank participants). Similar to the results of the original GEE analysis for all the rank groups, significant main effects of (normalized) EV and of Low-Rank (vs. High-Rank), but no interaction between (normalized) EV and Low-Rank (vs. High-Rank) were observed. These results indicate that presentation of low ranks generally increased the tendency to play, consistent with the results of the analyses described above at least to a certain extent, and also that presentation of low ranks did not change the sensitivity to EVs.

**Table 3:** Effects of EV and ranks on playing/passing.

Variable	β	Standard error	*z*-value	*p*-value	95% CI	
					Lower limit	Upper limit
Trials	-2.0442 × 10^-4^	0.0011	-0.1915	0.8481	-0.0023	0.0019
Normalized EV	0.6734	0.0846	7.9549	1.7764 × 10^-15^	0.5075	0.8393
High-Rank	-0.1249	0.2073	-0.6025	0.5469	-0.5313	0.2814
Low-Rank	0.4870	0.1768	2.7537	0.0059	0.1404	0.8336
Normalized EV × High- Rank	0.0777	0.1513	0.5137	0.6075	-0.2188	0.3742
Normalized EV × Low-Rank	-0.0313	0.1603	-0.1955	0.8450	-0.3454	0.2828
Constant	0.5295	0.1285	4.1194	3.7983 × 10^-5^	0.2776	0.7815

*GEE estimate of α = 0.21625*.

**Table 4:** Effects of EV and low, vs. high, ranks on playing/passing.

Variable	β	Standard error	*z*-value	*p*-value	95% CI	
					Lower limit	Upper limit
Trials	7.6755 × 10^-4^	0.0015	0.4987	0.6180	-0.0022	0.0038
Normalized EV	0.7517	0.1255	5.9898	2.1007 × 10^-9^	0.5057	0.9977
Low-Rank (vs. High-Rank)	0.6123	0.2140	2.8610	0.0042	0.1928	1.0318
Normalized EV × Low-Rank (vs. High-Rank)	-0.1076	0.1855	-0.5801	0.5618	-0.4711	0.2559
Constant	0.3410	0.1865	1.8287	0.0675	-0.0245	0.7065

*GEE estimate of α = 0.23053*.

As described in the Additional Notes in the Section “Materials and Methods,” for ethical and practical reasons, High-Rank participants in the roulette task were presented with low ranks in the experiment preceding the roulette task and vice versa. The observed differences in the behavior in the roulette task between the rank groups could thus potentially be effects of rank presentation in the preceding task, especially given that winning/losing causes changes in physiological states such as the level of testosterone, which could in turn affect behavior (Mazur and Booth, 1998; Casto and Edwards, 2016). In order to test this possibility, we compared the behavior between No-Rank participants who were presented with high ranks in the preceding task (18 and 17 participants in the first and second session of the preceding task, respectively) and No-Rank participants presented with low ranks previously (16 and 10 participants in the first and second session, respectively). The play ratio for the non-positive roulette wheels (excluding the initial ten trials) did not significantly differ between these groups (Welch’s *t*-tests: *p* = 0.7542 for low vs. high ranks in the first session; *p* = 0.9080 for low vs. high ranks in the second session; *p* = 0.6531 for low vs. high ranks in either session). We further conducted GEE analysis with covariates of trials (11th to 120th), normalized EVs, Low-Rank in the preceding task in both the first or second session (dummy-coded), and a constant term specified in the model and assuming the Markov correlation structure. This analysis revealed no significant effect of the Low-Rank in the preceding task [*p* = 0.7206 (robust covariance matrix-based results)]. These results indicate that the observed differences between the rank groups in the roulette task were likely to be caused by rank presentation in the roulette task itself rather than by rank presentation in the preceding task.

## Discussion

The present results demonstrate that awareness of one’s rank amongst peers affects the tendency to make risky choices in adolescent males in the natural classroom environment. Previous studies have shown that adolescents tend to take more risks when they are surrounded or observed by peers than when they are alone, presumably because the presence of peers constitutes a social reward that can bias decision making (Gardner and Steinberg, 2005; Chein et al., 2011; Smith et al., 2014). Our results support this, and furthermore show that this increased risk-taking is further enhanced by perception of low ranks amongst peers. We originally hypothesized that presentation of low ranks, or high ranks, or both would increase the ratio of choosing ‘play’ especially for roulette wheels with non-positive EVs. Our analyses on the play ratio are consistent with this hypothesis, indicating that only low ranks enhance the play ratios. The GEE analyses, showing that presentation of low ranks increased the tendency to play, also support our hypothesis at least to a certain extent, while it was also suggested that presentation of low ranks did not change the sensitivity to EVs.

Recent work has delved into the details of peer interactions, showing that peer observation and peer advice have distinguishable effects on risky decision making (Haddad et al., 2014). Peer competition is also a typical type of peer interaction, and recent work has suggested that winning/losing in a competition is an intrinsic social reward/punishment (van den Bos et al., 2013a,b). However, the effects of peer competition on adolescent risk-taking have remained elusive and our results provide a clue toward answering this question, at the same time demonstrating the utility of smartphone technology to explore these heretofore types of hard to reach questions.

A body of studies has examined the effects of status/hierarchy amongst peers on aggressive/antisocial behavior in adolescents and children (Coie et al., 1982; Xie et al., 2002; Prinstein and Cillessen, 2003), finding that aggressive behavior is associated with high popularity, low likability, and also with low popularity to some extent (i.e., non-linear relation to popularity) (Prinstein and Cillessen, 2003). The present study has implications to these lines of research in two ways. First, while previous studies have achieved classifications of types of aggressive behaviors, we focused on a specific type of elementary cognitive function, namely, decision making when faced with risky options. How the tendency for risk-taking relates to the different types of aggressive/antisocial behavior awaits future research. Second, whereas previous studies examined the effects of existing, long-term status, we introduced and manipulated temporary statuses that were independent of existing hierarchies. It was expected that effects would be observed given that adolescents might be sensitive to temporary statuses due to their potentially destabilizing effects on existing hierarchies, and our results support this expectation.

A recent study (Zhu et al., 2016) examined how winning/losing in a task with inter-group competition affects the tendency for risk-taking in subsequent choice opportunities in children. Intriguingly, compared to winners or control participants who performed the task without inter-group competition, it was observed that losers became risk-averse. This differs from our results, and this might be explained by several important differences between the two studies, including the age of participants (adolescents aged 14–15 in the current experiment vs. children 2–9 years old in Zhu et al., 2016), inter-individual vs. inter-group competition, multiple choices with risky options made during and affecting the competition vs. a single choice made after the competition, and whether the ranks (winning/losing) presented were fake or real. Exploring how these factors relate to risk-taking tendencies would be important in clarifying the relationships between risky behavior in adolescents and peer competition.

In our experiments, even participants in the No-Rank group were surrounded by their peers, and thus a basic effect of peer presence on risk-taking might apply to them too. Consistent with this, the play ratio for roulette wheels with negative EVs in participants not presented ranks was around 30–40% at the median, which is comparable to or even greater than the ratio observed in a previous study where peer presence was a factor (Smith et al., 2014) (note: participants in our study were 1–2 years younger than participants in the previous study, 14–15 vs. 15–17 years old). The interpretation of this is that awareness of one’s own position amongst peers affects risky behavior in addition to the basic peer effect.

The reason why awareness of being in a losing position (Low-Rank) induced more risk-taking than awareness of being in a winning position (High-Rank) needs further discussion. Awareness of low rank may be taken as a social punishment, and may make one eager to compensate by obtaining immediate reward even when the level of risk is high. On the other hand, those who are winning might be socially satisfied and as such not want immediate reward so much unless it comes with low risk. They may wish to retain their high position and thus would not make choices which could jeopardize their high rank. Looking at the behavioral economics literature, the prospect theory account of risk-taking might provide a good interpretation for our results (Kahneman and Tversky, 1979). Prospect theory implies that misperceptions or uneven weighting of probability contribute to risky decision making. The misperceptions of probability have been suggested (Snowberg and Wolfers, 2010) to account for the favorite-longshot bias (Griffith, 1949). It has further been discussed (Thaler and Ziemba, 1988) that the favorite-longshot bias would tend to become more prominent for the last races of the day (McGlothlin, 1956) possibly due to loss aversion (Kahneman and Tversky, 1979): “*Bettors on average are losing toward the end of the day. They would like to go home a winner, but do not want to risk losing much more money* (quoted from page 171 of Thaler and Ziemba, 1988),” although loss aversion was not evident in recent data (Snowberg and Wolfers, 2010). The increase of the play ratio for the non-positive roulette wheels in the Low-Rank group in our experiment could have a similar origin. Specifically, it is possible that the longshot bias was manifested particularly in the participants who were aware of being in low ranking positions, although the probabilities to win with the non-positive roulette wheels were not that low and whether the overweighing of low probability actually occurred seems unclear.

It is also possible that low-rankers made riskier choices because they believed that attempting ‘longshots’ was the only way to catch up to their competitors, even knowing that this type of response was irrational in terms of point maximization. By the same token, it seems as if participants put greater emphasis on the ‘social reward’ aspect of low rank than the ‘point reward’ on each trial, causing in a bias toward a ‘longshot’ mentality. Potentially contributing factors include how participants estimated the rationality of the opponents and how they estimated the number of remaining trials and the point differences between themselves and the opponents. Also possibly involved was how important the different ranks were to them (e.g., strongly preferring only 1st place or a linear rank preference), and how they differentially preferred strategies of playing for their own point-gain as compared to passing and waiting for the opponents to lose points on their own. More elaborate experimental designs and analyses will be needed to dissociate these possible factors. Nonetheless, effects of the awareness of ranks on risk-taking related to any of these factors can contribute to the observed problematic risky behaviors in adolescents, and the present study demonstrated that the awareness of even temporary ranks indeed has effects on risk-taking.

Besides the abovementioned issues, there are several limitations in the present study. First, here we examined only adolescents, and comparisons with adults and children would surely be interesting and informative. While conducting comparable experiments for adults would not be easy given that there are no exact counterparts to classmates or classrooms (the office environment, while potentially applicable, presents many different dynamics and variables), it may be possible for young adults, i.e., college students. Second, all participants were males; comparison with females would also be interesting especially given the suggested relationships between testosterone and competitive behavior (Mazur and Booth, 1998; van den Bos et al., 2013a; Casto and Edwards, 2016). Third, all the participants in the present study were students at the same junior high school, and results might reflect specific characteristics of that school. Fourth, participant ranks were not observed by others in real time (although students compared their results after the experiment without prompting). Even so we observed significant effects; it would be interesting to examine situations in which ranks can be seen by others. Fifth, the present work examined temporary status, i.e., ranking among peers during a short competition. Whether established (or slowly changing) hierarchies show similar or different effects still remains an open question. Sixth, it bears repeating that all gambles were a mixture of gains, losses and zeroes. As gains and losses (and zeroes) may be qualitatively different (Kahneman and Tversky, 1979; Weller et al., 2011; Yechiam and Hochman, 2013), mixing the gambles potentially had specific effects in the current study. Seventh, we did not assess pubertal timing, which potentially affects the tendency for risky behavior. Finally, only behavioral data were examined. Measurements or manipulations of neural and hormonal systems have the potential to provide insights into underlying physiological mechanisms.

To our knowledge, this study is one of the first demonstrations of the effectiveness of using smartphone devices experimentally in a school setting to reach the natural environment of adolescents and provide acute manipulations of peer interactions. The present results demonstrate the promise of smartphones and mobile technology as tools to examine peer interactions in the classroom. Taking advantage of this technology, we revealed that awareness of being in a losing position amongst peers induces more risk-taking than awareness of being in the lead or complete non-awareness of ranks in adolescent males. Our results have important implications for the development of possible methods of intervention. Specifically, in order to prevent risky behavior in adolescents, not only direct interventions, but also interventions mitigating the feelings of inferiority in competitive situations or hierarchies among peers may be effective.

## Author Contributions

JF, KN, and KM designed the work. JF, KN, KY, YY, and KM developed the smartphone system for the experiment. JF and KN conducted the experiment. JF, KN, SN-O, and KM analyzed the data. All the authors interpreted the data. JF, SN-O, and KM drafted the work. All the authors revised the work.

## Conflict of Interest Statement

The authors declare that theresearch was conducted in the absence of any commercial or financial relationships that could be construed as a potential conflict of interest.

## Footnotes

1 https://www.r-project.org/

2 https://www.jstatsoft.org/article/view/v025i14
